# Oestrogen blocks the nuclear entry of SOX9 in the developing gonad of a marsupial mammal

**DOI:** 10.1186/1741-7007-8-113

**Published:** 2010-08-31

**Authors:** Andrew J Pask, Natalie E Calatayud, Geoff Shaw, William M Wood, Marilyn B Renfree

**Affiliations:** 1 Department of Molecular and Cellular Biology, University of Connecticut, Storrs, CT 06260, USA; 2 Department of Zoology, The University of Melbourne, Melbourne, Victoria 3010, Australia

## Abstract

**Background:**

Hormones are critical for early gonadal development in nonmammalian vertebrates, and oestrogen is required for normal ovarian development. In contrast, mammals determine sex by the presence or absence of the *SRY *gene, and hormones are not thought to play a role in early gonadal development. Despite an XY sex-determining system in marsupial mammals, exposure to oestrogen can override *SRY *and induce ovarian development of XY gonads if administered early enough. Here we assess the effect of exogenous oestrogen on the molecular pathways of mammalian gonadal development.

**Results:**

We examined the expression of key testicular (*SRY*, *SOX9*, *AMH *and *FGF9*) and ovarian (*WNT4*, *RSPO1*, *FOXL2 *and *FST*) markers during gonadal development in the marsupial tammar wallaby (*Macropus eugenii*) and used these data to determine the effect of oestrogen exposure on gonadal fate. During normal development, we observed male specific upregulation of *AMH *and *SOX9 *as in the mouse and human testis, but this upregulation was initiated before the peak in *SRY *expression and 4 days before testicular cord formation. Similarly, key genes for ovarian development in mouse and human were also upregulated during ovarian differentiation in the tammar. In particular, there was early sexually dimorphic expression of *FOXL2 *and *WNT4*, suggesting that these genes are key regulators of ovarian development in all therian mammals. We next examined the effect of exogenous oestrogen on the development of the mammalian XY gonad. Despite the presence of *SRY*, exogenous oestrogen blocked the key male transcription factor SOX9 from entering the nuclei of male somatic cells, preventing activation of the testicular pathway and permitting upregulation of key female genes, resulting in ovarian development of the XY gonad.

**Conclusions:**

We have uncovered a mechanism by which oestrogen can regulate gonadal development through the nucleocytoplasmic shuttling of SOX9. This may represent an underlying ancestral mechanism by which oestrogen promotes ovarian development in the gonads of nonmammalian vertebrates. Furthermore, oestrogen may retain this function in adult female mammals to maintain granulosa cell fate in the differentiated ovary by suppressing nuclear translocation of the SOX9 protein.

See commentary: http://www.biomedcentral.com/1741-7007/8/110

## Background

During vertebrate evolution, there has been a distinct switch from a strong hormonal influence on sex determination to a genetic switch mechanism. In nonmammalian vertebrates, oestrogen administration to male embryos, or blocking oestrogen production in female embryos, results in sex reversal [1]. In contrast, in mice, oestrogen does not affect the early development of either ovaries or testes, and in its absence, gonadal development proceeds normally until after birth [2]. Although the role of oestrogen in determining gonadal fate differs between vertebrates, the downstream pathways of gonadal differentiation are highly conserved. In particular, *SOX9*, the key regulator of testicular differentiation, appears to have similar functions in all vertebrates [3], starting with cytoplasmic expression in the indifferent gonad, but translocating to the nucleus in males to activate the testicular pathway [4-6]. In oestrogen-deficient female mice (made by deletion of one or both oestrogen receptor genes or of the Cyp19A1 gene that encodes the aromatase enzyme), germ cells are gradually lost from pubertal ovaries concomitant with remodeling of the somatic tissue. There is also transdifferentiation of the ovarian granulosa cells to form testicular Sertoli-like cells that express *SOX9 *and align in structures resembling testicular cords [7]. This phenotype can be reversed by administration of oestrogen, causing the somatic cells to reform granulosa cells and resulting in an ovarian architecture and suppression of *SOX9 *[7]. These data show that although oestrogen is not required for early ovarian development in mice, it is critical for maintaining ovarian somatic cell fate in the differentiated gonad.

How and why oestrogen became dissociated from sex determination in mammals is unknown. A genetically controlled sex determination mechanism may have evolved in mammals to protect the young developing *in utero *from the effect of large quantities of oestrogen in the fetoplacental unit [8,9]. However, oestrogen receptor expression is maintained in the indifferent mammalian gonad [10,11] despite their apparent lack of function, and exposure of gonads to exogenous oestrogen activates the translocation of the receptors to the nuclei of indifferent gonadal cells [12]. Not surprisingly, the mammalian placenta is highly effective in metabolizing hormones before they reach the developing fetus [13], protecting it from maternal influences. Marsupial mammals are born with undifferentiated gonads that undergo gonadal sex determination postnatally [14], allowing daily administration of oestrogen directly to developing male young from before the testis pathway is initiated. Oestrogen treatment of XY young in the opossum and in the tammar wallaby directs the development of the indifferent gonads towards an ovarian differentiation fate [15-21], including entry of the XY germ cells into meiosis, despite the presence of a Y-chromosome and *SRY *[17-19,21].

The sex-reversing effects of exogenous oestrogen on the marsupial XY gonad are time-dependent [17,18], just as there is a critical time window of *Sry *action in the mouse [22]. Complete ovarian formation with germ cells arrested in meiosis was seen only in animals that were born about 1 day prematurely in a 26.5-day pregnancy in the tammar [18,21] and one half to 1 day prematurely in the 13.5-day gestation in the opossum [17]. Treatment of animals born after a full-term pregnancy with the same dose of oestrogen did not affect testicular development and resulted in only minor disruption of the testis in both species [17-19].

These findings suggest that there is a critical point in development when the XY gonad becomes irreversibly committed to a testicular fate. In the tammar, this coincides with the first morphological signs of Sertoli cell development and seminiferous cord formation, which occurs after birth from a normal full-term pregnancy [14]. To promote ovarian development, oestrogen must be administered before the somatic cell lineage has initiated Sertoli cell development. Early experiments treating opossums with large amounts of androgens did not affect gonadal development of male or female young [15,16]. Similarly, in the tammar, testosterone administration to male pouch young had no visible effect on testicular structure, although oestrogen given after testes development was initiated did cause a small degree of testicular dysgenesis [20]. Identical findings have also been reported in nonmammalian vertebrates in which administration of oestrogen to the indifferent gonad can actively induce ovarian development, independent of genetic or environmental sex-determining factors [1,23], whereas exogenous androgen treatment does not affect testicular development [23]. The effects of oestrogen are mediated through the oestrogen receptors (ERs) in the gonad [2]. Treatment of the tammar XY gonads at day 25 of gestation with oestrogen in culture causes the oestrogen receptors to move from a cytoplasmic to nuclear localization within the cell, consistent with their activation and ability to affect gene transcription in an oestrogen-dependent manner [12]. The nuclear translocation of both receptor proteins in the presence of exogenous oestrogen in culture confirms the ability of the receptors to respond functionally to the ligand, even at early developmental stages [12]. Thus the observed effects of exogenous oestrogen are oestrogen-, and not steroid-, specific.

The genes controlling sex determination are conserved between eutherian and marsupial mammals. *SRY *and *SOX9 *are expressed in the developing Sertoli cells of the XY eutherian and marsupial testis [4,24,25]. These cells direct the development of the testicular architecture and secrete AMH, a hormone necessary for the regression of the Müllerian ducts [26-28]. In the absence of *SRY *in developing XX gonads, *SOX9 *levels are significantly lower [4,25], *AMH *is not activated and the Müllerian ducts remain intact [26,27]. FGF9 may also be important for Sertoli cell specification [29] and shares an antagonistic relationship with an ovarian differentiation gene, *WNT4 *[30]. Other key ovarian development genes include *FOXL2, RSPO1 *and *FST*, which are upregulated in the somatic cells of the early developing ovary in mice and humans [31-33]. Disruption of each gene dramatically affects ovarian differentiation and can lead to varying degrees of testicular development in XX mammals [31-33].

To determine the molecular changes occurring during oestrogen -induced sex reversal of XY gonads, we investigated the expression of key male and female differentiation factors (*SRY*, *SOX9*, *AMH *and *FGF9 *and key ovarian markers *FOXL2, FST, RSPO1 *and *WNT4*) in our model marsupial, the tammar wallaby, during the critical period of gonadal differentiation and after oestrogen-induced sex reversal. Here we show that exposure of the indifferent XY gonad to oestrogen resulted in suppression of *SRY *and *AMH *and upregulation of *FOXL2 *and *WNT4*, thus diverting the male developmental pathway into the female one. The *SOX9 *gene was not downregulated by oestrogen exposure during XY ovarian development, but SOX9 protein was prevented from entering the nucleus of the supporting cells in the presence of oestrogen and therefore could not activate the male developmental program. We hypothesise that this novel function of oestrogen in modulating the nucleocytoplasmic localization of SOX9 may reflect an ancestral primordial mechanism by which oestrogen mediates sex determination in nonmammalian development. However, oestrogen retains an important function in adult female mammals by maintaining granulosa cell fate in the differentiated ovary by suppressing nuclear localization of the SOX9 protein.

## Results

### Molecular development of the marsupial gonad mirrors that of the eutherian gonad

**Figure 1:**
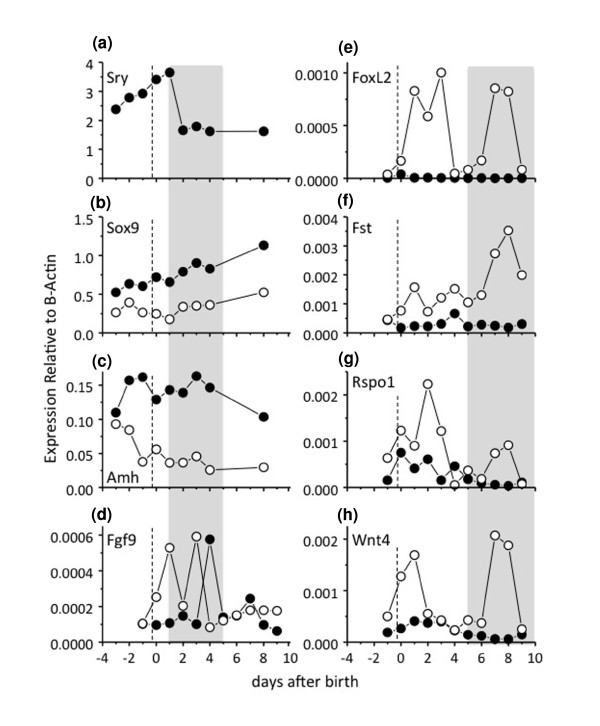
**Quantitative mRNA expression for *SRY*, *SOX9*, *AMH, FGF9, FOXL2, FST, RSPO1 *and *WNT4 *during normal male (solid circles) and female (open circles) gonadal differentiation**. Dotted line indicates day of birth, grey shading in a-d indicates the period of testis patterning and grey shading in e-h indicates the window of ovarian patterning. The critical period of testicular differentiation occurs between day 25 of gestation and day 2 postpartum, and that of ovarian differentiation between days 6-10 postpartum. *P *values from *t*-tests conducted to examine whether expression levels of the various genes were significantly different between males and females at each data point are shown in Additional file 1. Raw data for the mean ΔCt and standard deviations for each data point are shown in Additional file 5.

We used quantitative polymerase chain reaction (qPCR) to examine the normal expression profiles of key male and female gonadal differentiation genes during early testicular and ovarian development from day 25 of gestation (-1 day relative to birth) through day 10 postpartum in the tammar wallaby (*n *≥ 5 per sex per stage). The molecular control of testicular development in the developing tammar was similar to that of the mouse and human, except that the first signs of sexually dimorphic expression of *SOX9 *and *AMH *preceded the peak in *SRY *expression and testicular differentiation. *SRY *(Fig. 1a) expression increased from day 24 of gestation (-2 days relative to birth) in XY gonads and reached a peak at day 1 postpartum (pp) around the time of testicular cord formation. *SRY *levels then fell sharply as in the mouse [34,35] and human [36]. In tammar males, *SOX9 *(Fig. 1b) rose in the gonad, initially in concert with *SRY*, but continued to increase after *SRY *expression fell, consistent with its suggested autoregulation [37]. *SOX9 *was significantly higher in males than in females from day 24 of gestation (*P *= 0.001; Additional File 1), 4-5 days before the onset of testis cord formation, consistent with its activation by *SRY *binding to the conserved testis-specific enhancer element *TESCO *in the *SOX9 *promoter, that is found in marsupials as well as in eutherians [3,37]. In female gonads, *SOX9 *levels declined relative to levels in the testes, presumably due to the absence of *SRY*. *SOX9 *levels were lowest in females at day 1-2 pp (the time of cord formation in testes) and remained consistently lower in the ovary throughout development. *AMH *expression (Fig. 1c) was identical at day 24 of gestation in XX and XY gonads, but became significantly lower in ovaries (*P *= 0.0002; Additional File 1) by day 26 of gestation, the day of birth. The sexual dimorphism in *AMH *expression is consistent with reduced *SOX9 *expression in XX compared to XY gonads by day 26 of gestation (*P *= 0.0001). *AMH *levels remained significantly lower in ovaries than in testes throughout the period of development examined. *FGF9 *expression (Fig. 1d) was unexpectedly higher in females than in males during very early testis cord formation (days 1-3 pp; *P *< 0.01) but reached peak expression above that seen in the ovary by day 4 pp (*P *< 0.01). This is concomitant with a drop in *WNT4 *expression at this stage (Fig. 1h), further supporting an antagonistic interaction between these two genes in the gonad [30]. *FOXL2 *(Fig. 1e) was significantly lower in males than in females over the entire period examined (*P *< 0.01), consistent with expression seen in the developing mouse gonads [38], which suggests a conserved early role for this gene in mammalian ovarian development. *FST *expression (Fig. 1f) was not significantly lower in developing testes than in ovaries during early development but became significantly upregulated in developing ovaries after day 5 pp (*P *< 0.004) during the time of ovarian differentiation. *RSPO1 *(Fig. 1g) was not significantly lower in males than in females during early gonadal development. However, on day 4 pp, *RSPO1 *levels dropped in females and were significantly higher in males (*P *< 0.007). After day 4 pp, levels dropped in males and increased in females during the key window of ovarian differentiation. *WNT4 *expression (Fig. 1h) was generally higher in females than in males but this was only significant after day 4 pp, again during the time of active ovarian differentiation, as seen for *FST *and *RSPO1*. *P *values were significantly different at almost all time points for *FOXL2*, *FST*, *AMH *and *SOX9 *(Additional File 1).

### Exogenous oestrogen causes development of ovarian structures in XY gonads

**Figure 2:**
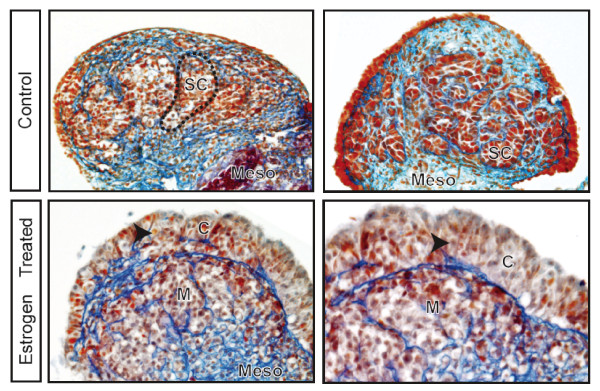
**Structure of control and oestrogen-cultured gonads**. Oestrogen-cultured XY gonads developed ovarian-like cortical and medullary structures with germ cells distributed at the periphery of the cortex (arrowhead), while control gonads developed normal testicular architecture with germ cells confined to testis cords. Gonad plus mesonephros (meso)complexes were sectioned at 8 μm and stained with Mallory's triple stain. Top: Gonads cultured in control media. Bottom: Gonads cultured in the presence of oestrogen. Control XY gonads have normal male development with the formation of seminiferous cords (SC; outlined by dashed line) delineated by a basal lamina (stained dense blue) with the germ cells contained within these structures. Oestrogen-treated XY gonads formed a cortex (C) which contained germ cells and a central medulla (M), resembling the normal ovarian architecture of equivalent stage XX gonads.

To verify that exogenous oestrogen would cause gonadal sex reversal in culture as it does *in vitro *[18], we examined the histological organization of the gonads from two control and two oestrogen-treated XY gonads after incubation in either control or oestrogen-supplemented media. Gonads were collected at day 25 of gestation, just before gonadal sex determination and birth. Gonads placed in control media and grown for 5 days had normal testicular architecture with the formation of seminiferous cords lined with Sertoli cells, encapsulating the germ cells (Fig. 2, top). Conversely, XY gonads cultured in the presence of oestrogen did not develop testicular cords and instead the gonads took on an ovarian appearance, with the formation of a clear demarcation between the cortex and medulla (Fig. 2, bottom).

### Exogenous oestrogen has a dramatic effect on the testicular differentiation pathway

**Figure 3:**
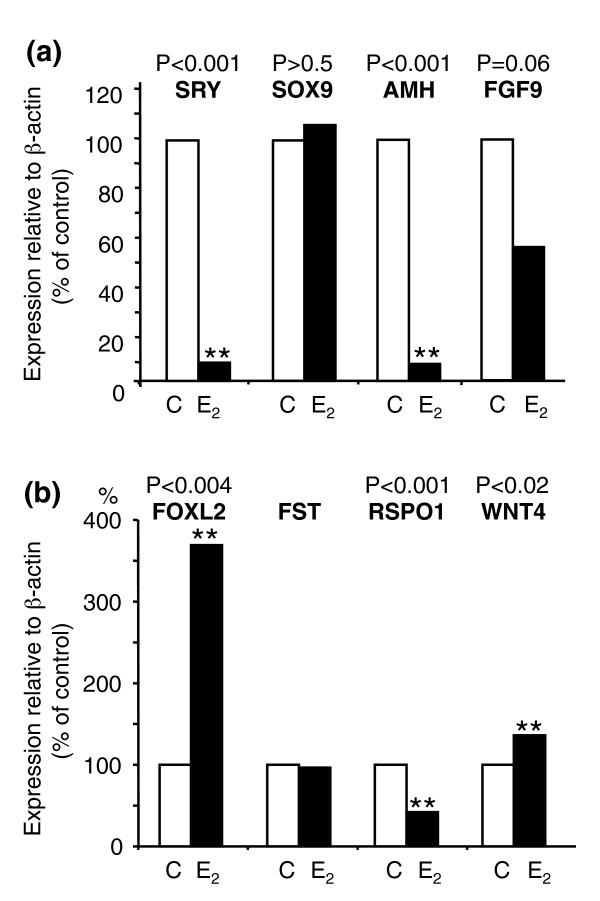
**Gene expression in XY oestrogen-treated gonads cultured from day 25 of gestation for 5 days**. Quantitative PCR for key male markers *SRY*, *SOX9*, *AMH *and *FGF9 *(a), and key female markers *FOXL2, RSPO1, WNT4 *and *FST *(b) during gonadal culture in control (open bars) and oestrogen-treated (black bars) gonads. Both *SRY *and *AMH *mRNA levels were significantly suppressed (*P *< 0.001) in the sex-reversed gonads. However, *SOX9 *mRNA levels were unchanged (*P *> 0.5). There was significant upregulation of the ovarian genes *FOXL2 *(*P *< 0.004) and *WNT4 *(*P *< 0.02) in sex-reversed gonads, while *FST *was unchanged and *RSPO1 *was significantly downregulated (*P *< 0.001) in the presence of oestrogen.

We next investigated the effect of sex reversal on the expression of key testicular genes in 10 pairs of null control and oestrogen-treated XY gonadal cultures by quantitative PCR. In the control gonads, the levels of *SRY*, *SOX9 *and *AMH *were comparable to those *in vivo*, demonstrating that the gonads developed normally in culture. However, the mRNA levels of both *SRY *(*P *< 0.001) and *AMH *(*P *< 0.001) were significantly reduced in XY gonads cultured in the presence of oestrogen (Fig. 3a, Additional file 2). *FGF9 *levels were also suppressed in oestrogen-treated gonads, although levels were not significant (*P *< 0.06; Additional file 2). In contrast, *SOX9 *mRNA levels did not differ significantly (*P *> 0.5) as a result of the treatment (Fig. 3a), despite the development of ovarian architecture. This was unexpected because *SOX9 *upregulation induces testis formation in eutherian mammals [39,40].

### SOX9 protein remains cytoplasmic in the presence of oestrogen

**Figure 4:**
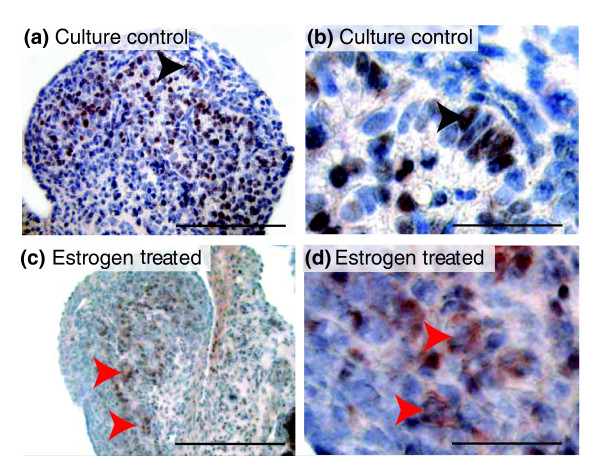
**The distribution of SOX9 protein in oestrogen-treated and control cultures**. Immunohistochemistry showing the subcellular distribution of SOX9 in control and oestrogen cultured XY gonads, using AEC+ chromogen red/brown staining (Dako) and counterstained with haematoxylin (blue). In the culture controls (a; magnified in b) localization was nuclear (black arrowhead) and confined to the Sertoli cells of the developing seminiferous cords. In contrast, in the oestrogen-cultured gonads (c; magnified in d), SOX9 was largely cytoplasmic (red arrowheads) and no or very few nuclei were stained. Scale bars are 100 μm in (a) and (b) and 25 μm in (c) and (d).

As a transcription factor, it is not just the level of SOX9, but also its subcellular localization that is essential for its function in initiating testicular development [4]. We therefore investigated SOX9 protein distribution using immunocytochemistry in two control and two oestrogen-treated XY gonads. In control XY gonads, SOX9 was almost entirely nuclear and restricted to the Sertoli cells of the developing testis, consistent with its distribution seen during normal *in vivo *development [25] (Figs. 4a and 4b). However, XY gonads cultured in the presence of oestrogen had diffuse SOX9 staining that was restricted to the cytoplasm of gonadal cells and did not enter the nuclei (Figs. 4c and 4d). Since SOX9 levels were sexually dimorphic before gonadal differentiation, we also investigated protein distribution at day 25 of gestation (the first day of culture). At this stage, SOX9 had a strong cytoplasmic distribution but had become nuclear in a subset of somatic cells (Additional file 3).

### Exogenous oestrogen activates the female differentiation pathway

In addition to the downregulation of testicular genes, oestrogen exposure and lack of nuclear SOX9 in the tammar sex-reversed XY gonads led to an upregulation of the key ovarian differentiation genes *FOXL2 *(*P *< 0.004) and *WNT4 *(*P *< 0.02) (Fig. 3b, Additional file 1), consistent with findings that both genes are critical early regulators of ovarian development [31,33,38]. In tammar sex-reversed gonads, *FGF9 *was downregulated in concert with *WNT4 *upregulation, supporting a conserved antagonistic interaction between these two genes in mammalian gonadal differentiation [30]. *RSPO1 *was significantly downregulated (*P *< 0.001) in the presence of oestrogen, consistent with the normal expression of this gene at day 4 pp in males and females, suggesting that at this stage of development, *RSPO1 *is not required for marsupial ovarian differentiation. *FST *mRNA expression was not significantly altered by oestrogen treatment (*P *> 0.5; Fig. 3b and Additional file 2).

## Discussion

Marsupials occupy a unique evolutionary niche and can provide important information on the evolution and conservation of mammalian developmental pathways. This is the first comprehensive study of the expression levels of key testicular and ovarian genes during gonadogenesis in any marsupial. During normal development in the tammar gonad, expression of key male development genes is similar to that seen in the mouse and human, except that *AMH *and *SOX9 *show sexually dimorphic gene expression before the peak in *SRY *expression at day 1 pp. Furthermore, *SOX9 *has already begun its nuclear translocation 3 days before the peak in *SRY *expression and the appearance of testicular cords. This may be the result of the long period of *SRY *expression seen in marsupials [24], similar to that of human XY gonads [36] but different from the ultrashort pulse seen in mice [34,35]. The key female development genes *FOXL2 *and *WNT4 *were upregulated in the XX gonad during the time of ovarian development (5-10 days pp). However, *RSPO1 *and *FST *expression was variable in the early gonad, and female-specific upregulation was seen only during the later stages of ovarian development. This observation suggests that these two genes act downstream of *FOXL2 *and *WNT4 *in the ovarian differentiation pathway, at least in the tammar.

Cultured XY control gonads had normal male expression profiles and formed testicular cords. However, XY gonads cultured in the presence of oestrogen failed to upregulate *SRY *and *AMH *expression and instead upregulated *FOXL2 *and *WNT4*. The oestrogen-exposed XY gonads also developed ovarian architecture, confirming that female development had occurred. Surprisingly, mRNA levels for *SOX9*, the key factor essential for testicular development, did not differ between control and treated XY gonads, despite ovarian development. To investigate this further, we examined the protein localisation of SOX9 in control and oestrogen-treated XY gonads. Since SOX9 is a transcription factor, nuclear localisation is essential to activate the male developmental program and *SOX9 *mutations that affect nuclear import result in XY sex reversal in humans [41,42]. In the mouse and human, the SOX9 protein is initially cytoplasmic in the indifferent gonad of both XX and XY fetuses, but becomes rapidly nuclear and upregulated only after the peak of *SRY *expression in testes, whereas in ovaries, Sox9 remains cytoplasmic and is lost [4,5]. In the clawed toad, *Xenopus*, SOX9 also remains cytoplasmic in developing ovaries but becomes nuclear under testis-promoting conditions in the absence of oestrogen and is upregulated [6,43]. However, in the presence of oestrogen under ovary-promoting conditions, SOX9 remains cytoplasmic and is downregulated.

In mice, *Sry *initiates *Sox9 *upregulation within pre-Sertoli cells. Sox9 then autoregulates to reach threshold levels that activate testicular development [44]. In the tammar, SRY had initiated *SOX9 *upregulation before the cultures began (Fig. 1; Additional file 1) and before the peak in *SRY *expression 3 days later. Thus oestrogen exposure at this time (day 25 of gestation) did not prevent the initial wave of *SOX9 *mRNA upregulation, but the protein was restricted to the cytoplasm of somatic cells, preventing its activation of the male pathway and likely its self-propagated upregulation. This observation explains the initiation of ovarian development in the treated XY gonads in this *in vitro *study and in the earlier *in vivo *studies [17,18,21]. Treatment of developing turtle gonads with oestrogen at stages of development before gonadal sex determination had occurred (and prior to the initial upregulation of *SOX9*) prevented *SOX9 *from becoming upregulated to equivalent male or even female levels [45]. These findings suggest that there is also a direct action of oestrogen in repressing *SOX9 *transcription in turtles, as we have observed in the tammar. Interestingly, in the turtle, SOX9 protein is nuclear in female gonads before sex determination. We see a similar nuclear localization in the indifferent gonad in the tammar wallaby (Additional file 3). We hypothesize that in the absence of hormones at early stages of gonadal development, SOX9 can feely translocate to the nucleus of somatic cells. In mammals, *SOX9 *expression is reinforced by SRY, upregulating it above threshold levels enabling it to autoregulate and initiate testicular development. In nonmammalian vertebrates, the oestrogen production could act to directly suppress SOX9 upregulation and promote ovarian development. Our results show that oestrogen restricts translocation of the SOX9 protein, forcing it to remain in the cytoplasm and prevent its self-propagated upregulation. Since *SOX9 *mRNA is never elevated above basal levels in the oestrogen-treated male turtle, the effect of oestrogen on its subcellular localization cannot be observed in their gonads. However, in our study, we administered oestrogen slightly later in development, just after the initial wave of *SOX9 *upregulation. Our results therefore highlight a mechanism by which oestrogen could prevent testicular development (Fig. 4). The exclusion of SOX9 from somatic cell nuclei of the oestrogen cultured gonads is further confirmed by the failure of *AMH *upregulation because in the mouse, Sox9 acts as a nuclear transcription factor to drive *Amh *expression [46].

In the absence of nuclear SOX9 in XY oestrogen-treated gonads, female development was initiated. *FOXL2 *was significantly upregulated, consistent with an early and essential role for this gene in initiating ovarian development in mammals. In goats, *FOXL2 *induces aromatase (*CYP19*) expression, leading to oestrogen production in the developing ovary [38]. This mechanism could potentially ensure a block in male development by excluding SOX9 nuclear entry. This mechanism could work in synergy with activated ERs to facilitate FOXL2 binding to TESCO, further suppressing *SOX9 *mRNA production as recently suggested in goats [38]. However, the oestrogen response elements contained within the mouse TESCO element [37] do not fall into the evolutionarily conserved regions identified in most vertebrates [3], suggesting that this mechanism of oestrogen action may not be as important as its role to prevent SOX9 from entering the nucleus.

Several lines of evidence demonstrate that oestrogen acts as a modulator of the subcellular localization of SOX9. Sox9 is produced at a low level in the somatic cells of the adult mouse ovary [47], but its self-propagated upregulation is somehow blocked. Our findings suggest that circulating oestrogen could prevent the nuclear translocation of SOX9 in differentiated ovarian somatic cells, thus preventing its own upregulation. In the absence of oestrogen in female aromatase-deficient knockout mice, Sox9 is upregulated in ovarian somatic cells, as it can presumably translocate to the nucleus to propagate its own transcription. However, when exogenous oestrogen is administered to aromatase-deficient mice, this effect is reversible and *Sox9 *is repressed [5,48]. This mechanism therefore explains how *Sox9 *upregulation is able to occur in the oestrogen-deficient mouse ovary and how it can be subsequently suppressed with somatic cell transdifferentiation in the presence of exogenous oestrogen. These findings suggest that in mammals, oestrogen may still be a critical factor regulating ovarian somatic cell fate maintenance by preventing SOX9 nuclear translocation.

This fundamental role of oestrogen can explain its sex-determining ability in nonmammalian vertebrates. SOX9 remains cytoplasmic in the developing ovaries of nonmammalian vertebrates in the presence of oestrogen [5]. However, in the absence of oestrogen, SOX9 becomes nuclear and testicular development is initiated [41]. Our findings suggest that this may be a universal action of oestrogen in all vertebrates.

How oestrogen prevents SOX9 from entering the nucleus is still unknown. There are several known pathways that control SOX9 nuclear entry, such as altered importin-β1 or calmodulin binding, both of which facilitate effective transport of SOX9 across the nuclear pore complex [41,42]. In addition, sumoylation and/or ubiquitination also affect the subcellular localization and transcriptional activity of SOX9 [49,50]. Oestrogen is known to directly affect sumoylation and the subcellular localization of the nuclear receptor coactivator family member 3 protein in breast cancer cell lines [51]. Oestrogen may work in a similar way in the gonad by modulating sumoylation and/or ubiquitination of the SOX9 protein, trapping it in the cytoplasm.

## Conclusions

**Figure 5:**
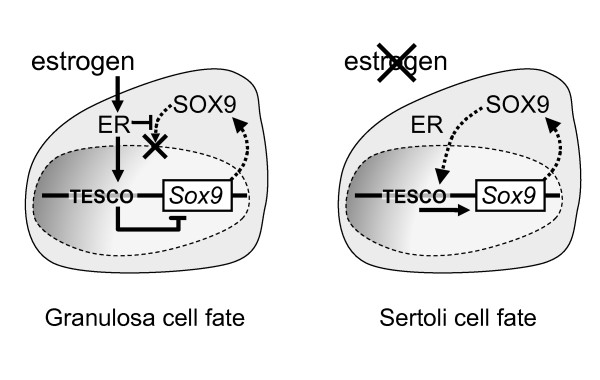
**Model for the role of oestrogen and the gonadal determination pathway**. In normal XY gonads, the somatic cells upregulate *SRY*, which in turn upregulates *SOX9*. SOX9 can then translocate to the nucleus and activate the expression of *AMH *to ensure normal male urogenital development. In the presence of oestrogen, the bipotential somatic cell is instead directed into the granulosa cell pathway. *SRY *fails to upregulate, and any SOX9 protein produced fails to enter the nucleus. In the absence of nuclear SOX9, *AMH *fails to upregulate and ovarian genes (*FOXL2 *and *WNT4*) are activated. Exogenous oestrogen therefore has the ability to direct XY bipotential somatic cells to a granulosa cell fate. Oestrogen may act in a similar manner to maintain granulosa cell fate in mature eutherian ovaries, preventing basal levels of SOX9 protein from translocating to the nucleus and propagating its own upregulation.

We have uncovered a mechanism using a marsupial model that provides a link between mammalian and nonmammalian vertebrate sex determination mechanisms (Fig. 5). We have shown that exogenous oestrogen can inhibit the nuclear translocation of SOX9, blocking testicular development in an XY mammalian gonad. As a result, ovarian differentiation occurred, concomitant with upregulation of *FOXL2 *and *WNT4 *(Fig. 3). We propose a model in which the role of oestrogen is to modulate the subcellular localization of SOX9 (Fig. 5). This could be an underlying and ancestral mechanism that controls ovarian development in nonmammalian vertebrates. In the absence of oestrogen in developing male fetuses, SOX9 would become nuclear and initiate testicular differentiation. However, in those nonmammalian vertebrates destined to become females, expression of aromatase and the production of oestrogen could prevent the nuclear translocation of SOX9, thus actively blocking testicular development. In mammals, the retention of this mechanism is not an evolutionary remnant of past function, but may have been retained to ensure that SOX9 nuclear entry is suppressed in the mature ovary to maintain granulosa cell fate.

## Methods

### Gonadal Culture

XY gonadal ridges (gonad and mesonephros taken together) were cultured in pairs from late-stage male tammar wallaby fetuses. The tammar wallaby gonadal ridge first develops on day 21 of the 26.5-day gestation period [14]. Seminiferous cords do not form in testes until day 2 pp, and ovarian differentiation is first seen around day 8 pp [14]. A total of 12 male fetuses were collected at day 25 of gestation, and pairs of gonads were isolated and placed, one into control media and the other into oestrogen media. Gonads were cultured for 5 days (120 hr) as previously described [12], with either Dulbecco's modified Eagle medium (DMEM)/10% fetal calf serum (FCS)/50 mg/ml ampicillin (control) or with the addition of estradiol benzoate (to a final concentration of 100 ng/ml). This dose was chosen to ensure sufficient oestrogen penetration into the cultured gonad. Estradiol benzoate was diluted in 100% ethanol at 1 mg/ml, and an equivalent amount of ethanol (without estradiol) was added to control cultures. At the conclusion of the culture period, gonads were either fixed in 4% paraformaldehyde and processed for histology or snap-frozen for molecular analyses.

### Histology

Two of the 12 control and two of the 12 oestrogen-treated XY cultured gonads were sectioned (8 μm) and stained with Mallory's triple stain to highlight the basal lamina (bright blue) in the developing gonads, according to standard methods.

### Immunohistochemistry

Anti-SOX9 antibody (Ab5535; Chemicon) was used at 1:100 dilution. Slides were then incubated with goat anti-rabbit biotin-conjugated secondary antibody (Molecular Probes). The avidin-biotin complex (ABC) kit (Vector Laboratories) was used according to the manufacturer's instructions, and antibody binding was visualized with the AEC+ chromogen (Vector Laboratories). Negative controls were performed as described above with the omission of the primary antibody and an equivalent amount of nonspecific IgG from the same species. No staining was observed in negative controls. Sections were counterstained with haematoxylin.

### RNA extraction and cDNA synthesis

RNA was extracted from gonads using the GenElute™ Mammalian Total RNA Miniprep Kit (Sigma) as per the manufacturer's instructions. Total RNA was DNase treated using the Turbo DNA free (Ambion) as per the manufacturer's instructions. Complementary DNA was produced using the Superscript III First Strand Synthesis System for reverse transcriptase (RT)-PCR (Invitrogen) to generate the first strand according to the manufacturer's instructions.

### Quantitative polymerase chain reaction

qPCR was carried out using the Quantitect real-time master mix (Qiagen) on a Bio-Rad CFX96 PCR machine. The genes of interest were normalized against β-actin expression using the ΔCT method described by Pfaffl [52] and taking primer efficiency into account. Standard *t*-tests (for sex reversal data; two-tailed, paired [gonad pairs; one cultured in oestrogen and the other in control media], for normal male profile; two-tailed, homoscedastic [comparing equal numbers of male and female gonads at different time points]) were used to compare the data sets using Microsoft Excel. For the normal gene expression profiles, data are shown relative to β-actin with individual gonads (*n *≥ 5) at each developmental time point for each sex. For gonadal culture, the remaining 10 gonadal pairs that were cultured were analyzed (20 gonads; 10 control and 10 oestrogen-cultured). The expression was normalized relative to β-actin. Expression levels in control gonads were designated 100%, and expression in the oestrogen-treated gonads expressed relative to control levels for each gene was examined. All real-time PCR reactions were subject to melt curve analysis and gel electrophoresis to check for single-product amplification. The primers chosen for amplification are listed in Additional file 4.

All experimental procedures conformed to Australian National Health and Medical Research Council (2004) guidelines and were approved by the University of Animal Experimentation Ethics Committees. Husbandry of tammar wallabies *(Macropus eugenii*) as well as aging and sexing of pouch young and fetuses were carried out as previously described [14].

## Authors' contributions

AJP and MBR conceived the study. All authors performed aspects of the research. AJP analyzed data. The manuscript was prepared by all authors.

## Supplementary Material

Additional file 1 **Statistical difference between male and female data points shown in Figure **1. *P *values (from *t*-*tests*) are listed for all data points shown in the normal expression profiles for *SOX9*, *AMH*, *FGF9*, *FOXL2*, *FST*, *RSPO1*, *WNT4*. Gene name is given in the top left hand corner of each data set. Column 2 shows the stage of development; d = day of gestation, D = postnatal day. Column 3 shows the *P *value from a two tailed, homoscedastic *t-test *(conducted in Microsoft Excel). Significant values (*P *≤ 0.05) are highlighted in yellow. The bottom row (combined M (male) v F (female)) shows the *P *values for the entire male versus female data set combined across the time period shown. *SOX9*, *AMH*, *FOXL2 *and *FST *showed the greatest difference between testes and ovaries. *WNT4 *was significantly higher in females than males over the entire period examined, but was not significantly higher at any given time point. Click here for file

Additional file 2 **Raw expression data for qPCR analysis of the oestrogen-treated and control culture gonads**. Graphs show expression relative to beta-actin (house keeping control gene) that shows high levels of expression. Small bars represent a small difference between target gene expression and that of beta-actin (thus, represent high expression values) while large bars show a greater difference between target gene levels and beta-actin (thus, representing low expression values). Error bars show one standard deviation either side of the mean. Click here for file

Additional file 3 **SOX9 distribution in the bipotential gonad**. SOX9 protein was examined in the day 25 fetal XY gonad to determine its distribution at the start of the culture period. At this time, SOX9 was already localized in the nuclei (black arrowhead) of many somatic cells, but some weak cytoplasmic staining (red arrowhead) also remained. Click here for file

Additional file 4 **Primers used**. Primers used in quantitative PCR reactions are listed in 5' to 3' orientation. Click here for file

Additional file 5 **Raw expression data for qPCR analysis of the normal gene profiles shown in Figure **1. Mean delta Ct values and the standard deviation (std-dev) for each data point shown in Figure 1. The housekeeping gene β-actin shows high levels of expression so most delta Ct values are negative. The left column represents data points from the male profile and the right column represents data points from the female profile. Gene names are indicated in the top left corner of each table. Graphed data is log transformed and shown relative to beta actin in Figure 1. Click here for file
