# Supplementary material for: Oestrogen blocks the nuclear entry of SOX9 in the developing gonad of a marsupial mammal
Source: BMC Biol. 2010 Aug 31;8:113. doi: 10.1186/1741-7007-8-113 (PMC2940779; doi:10.1186/1741-7007-8-113)
Supplement: Additional file 1 — Statistical difference between male and female data points shown in Figure 1. P values (from t-tests) are listed for all data points shown in the normal expression profiles for SOX9, AMH, FGF9, FOXL2, FST, RSPO1, WNT4. Gene name is given in the top left hand corner of each data set. Column 2 shows the stage of development; d = day of gestation, D = postnatal day. Column 3 shows the P value from a two tailed, homoscedastic t-test (conducted in Microsoft Excel). Significant values (P ≤ 0.05) are highlighted in yellow. The bottom row (combined M (male) v F (female)) shows the P values for the entire male versus female data set combined across the time period shown. SOX9, AMH, FOXL2 and FST showed the greatest difference between testes and ovaries. WNT4 was significantly higher in females than males over the entire period examined, but was not significantly higher at any given time point. [file 1741-7007-8-113-S1.PDF]

FOXL2	d26	0.0334
	D0	0.2345
	D1	<0.0001
	D2	0.0035
	D3	0.0037
	D4	0.0151
	D5	0.0640
	D6	0.0300
	D7	0.0003
	D8	0.0014
	D9	0.0047
Combined M v F		0.0415

AMH	d24	0.4262
	d25	0.0468
	d26	0.0003
	D0	0.1096
	D1	0.0092
	D2	<0.0001
	D3	0.0036
	D4	0.0003
	>D8	0.0204
Combined M v F		<0.0001

FST	d26	0.9166
	D0	0.1738
	D1	0.0199
	D2	0.3102
	D3	0.1054
	D4	0.0752
	D5	0.0027
	D6	0.0049
	D7	0.0025
	D8	0.0100
	D9	0.0018
Combined M v F		<0.0001

SOX9	d24	0.0011
	d25	0.0045
	d26	0.0001
	D0	0.0004
	D1	0.2045
	D2	0.0644
	D3	0.0296
	D4	0.0050
	>D8	0.0498
Combined M v F		<0.0001

RSPO1	d26	0.1094
	D0	0.6279
	D1	0.5081
	D2	0.1054
	D3	0.1643
	D4	0.0070
	D5	0.5922
	D6	0.5054
	D7	0.1220
	D8	0.5488
	D9	0.7245
Combined M v F		0.1998

FGF9	d26	0.9432
	D0	0.0650
	D1	0.0098
	D2	0.6811
	D3	0.0139
	D4	0.0148
	D5	0.8339
	D6	0.9812
	D7	0.6545
	D8	0.5587
	D9	0.0999
Combined M v F		0.1148

Wnt4	d26	0.2468
	D0	0.0583
	D1	0.1478
	D2	0.7643
	D3	0.9401
	D4	0.9437
	D5	0.1750
	D6	0.1587
	D7	0.0612
	D8	0.0759
	D9	0.4639
Combined M v F		0.0059
