# Supplementary figures and images for: Oestrogen blocks the nuclear entry of SOX9 in the developing gonad of a marsupial mammal
Source: BMC Biol. 2010 Aug 31;8:113. doi: 10.1186/1741-7007-8-113 (PMC2940779; doi:10.1186/1741-7007-8-113)

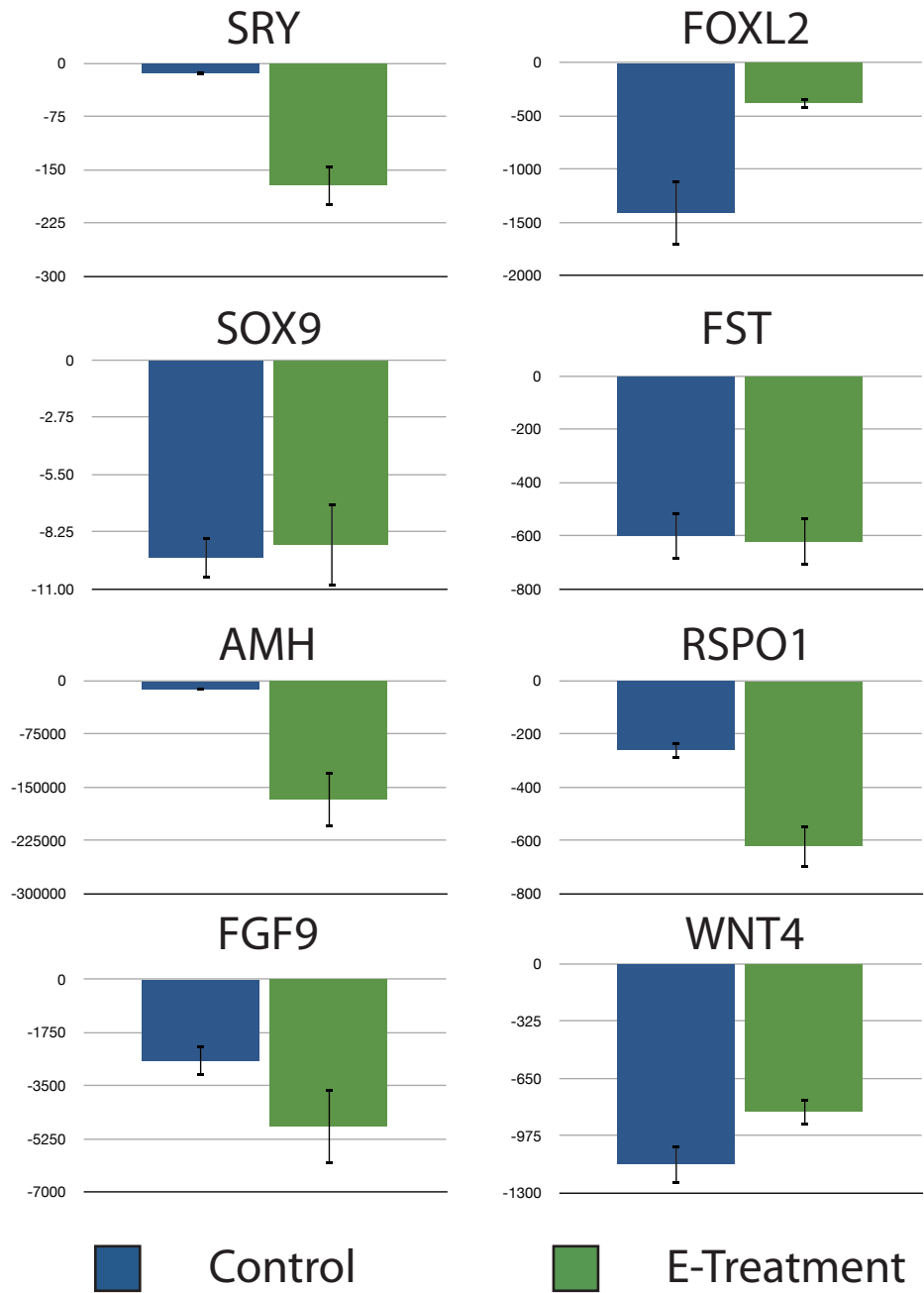

Supplement: Additional file 2 — Raw expression data for qPCR analysis of the oestrogen-treated and control culture gonads. Graphs show expression relative to beta-actin (house keeping control gene) that shows high levels of expression. Small bars represent a small difference between target gene expression and that of beta-actin (thus, represent high expression values) while large bars show a greater difference between target gene levels and beta-actin (thus, representing low expression values). Error bars show one standard deviation either side of the mean. [file 1741-7007-8-113-S2.PDF]

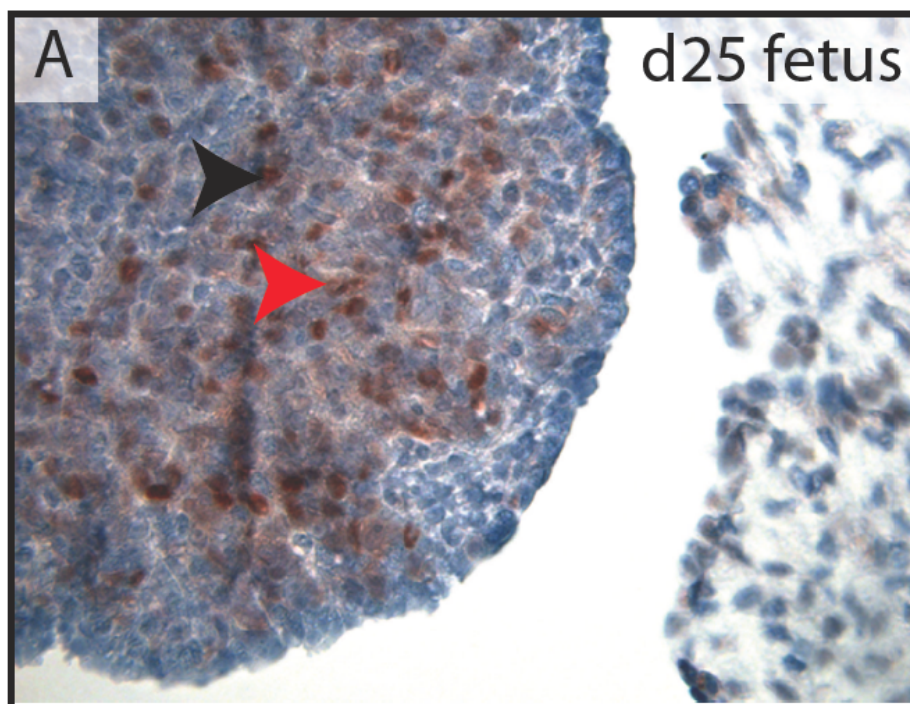

Supplement: Additional file 3 — SOX9 distribution in the bipotential gonad. SOX9 protein was examined in the day 25 fetal XY gonad to determine its distribution at the start of the culture period. At this time, SOX9 was already localized in the nuclei (black arrowhead) of many somatic cells, but some weak cytoplasmic staining (red arrowhead) also remained. [file 1741-7007-8-113-S3.PDF]
