# Supplementary material for: Oestrogen blocks the nuclear entry of SOX9 in the developing gonad of a marsupial mammal
Source: BMC Biol. 2010 Aug 31;8:113. doi: 10.1186/1741-7007-8-113 (PMC2940779; doi:10.1186/1741-7007-8-113)
Supplement: Additional file 4 — Primers used. Primers used in quantitative PCR reactions are listed in 5' to 3' orientation. [file 1741-7007-8-113-S4.PDF]

Gene name	Direction	Sequence (5'-3')
β–Actin	forward	TTGCTGACAGGATGCAGAAG
β–Actin	reverse	AAAGCCATGCCAATCTCATC
AMH	forward	CCTGAGGGTGGTGGGGGGTCT
AMH	reverse	AGCGGGTATGGTGTGGAGTCA
SOX9	forward	TGCGAGTCAATGGCTCTAGCAA
SOX9	reverse	CTCCTCCGAGGTTGGTATTTGT
SRY	forward	TTGAGTCCGTGAAAAGTGGGT
SRY	reverse	TTGTGAATCTGCCACGCTTGTC
FGF9	forward	ACAGCAGATTTGGGATCCTG
FGF9	reverse	TCCAGTGTCCACGTGTTTGT
Wnt4	forward	GAAACCGACGGTGGAAC
Wnt4	reverse	AGGAGATGGCATAGACGAA
FST	forward	ATCCCTTGCAAAGAAACGTGT
FST	reverse	TTCCAACTCTGGCTGCTCTT
FOXL2	forward	ACTCGTACGTGGCGCTTATC
FOXL2	reverse	GAGGCACCTTGATGAAGCAT
RSPO1	forward	CAGAGGCAGATCAGCACAGA
RSPO1	reverse	CAAAAATTGTGGCTGAAGCA
