# Supplementary material for: Oestrogen blocks the nuclear entry of SOX9 in the developing gonad of a marsupial mammal
Source: BMC Biol. 2010 Aug 31;8:113. doi: 10.1186/1741-7007-8-113 (PMC2940779; doi:10.1186/1741-7007-8-113)
Supplement: Additional file 5 — Raw expression data for qPCR analysis of the normal gene profiles shown in Figure 1. Mean delta Ct values and the standard deviation (std-dev) for each data point shown in Figure 1. The housekeeping gene β-actin shows high levels of expression so most delta Ct values are negative. The left column represents data points from the male profile and the right column represents data points from the female profile. Gene names are indicated in the top left corner of each table. Graphed data is log transformed and shown relative to beta actin in Figure 1. [file 1741-7007-8-113-S5.PDF]

Male

FGF9	std-dev	mean
d26	0.4515736	-13.25358
D0	0.17280295	-13.335425
D1	0.34539867	-13.194329
D2	1.59187223	-12.729459
D3	0.75948874	-13.269704
D4	0.80238564	-10.761166
D5	1.10706918	-12.818702
D6	1.30831804	-12.71917
D7	1.71917035	-12.348509
D8	1.26964437	-13.330346
D9	0.82584041	-13.9557

FOXL2	std-dev	mean
d26	1.20316541	-17.971147
D0	0.97102698	-14.6534
D1	0.72583018	-17.568629
D2	0.39415507	-17.22261
D3	1.21403555	-18.115058
D4	2.13888458	-18.263422
D5	2.12660484	-20.274768
D6	4.28571359	-19.213148
D7	1.73756231	-22.012437
D8	2.23433488	-22.782417
D9	2.48644446	-20.050425

FST	std-dev	mean
d26	0.2773608	-11.161775
D0	0.23522742	-12.486373
D1	0.84215406	-12.074264
D2	0.59463904	-12.138626
D3	1.07873679	-11.661724
D4	0.80793523	-10.55725
D5	0.50853633	-12.157233
D6	0.86456654	-11.792337
D7	0.90940512	-12.885268
D8	0.72901211	-12.402697
D9	1.00437945	-11.691355

RSPO1	std-dev	mean
d26	2.29548444	-12.63496
D0	2.53459062	-10.380764
D1	1.04702781	-11.248829
D2	0.41973442	-10.686121
D3	2.34447965	-12.644687
D4	1.14230214	-11.086279
D5	2.33565449	-12.439699
D6	2.34496451	-13.415027
D7	2.49298688	-14.588494
D8	1.28028664	-14.664054
D9	2.52511804	-13.167488

WNT4	std-dev	mean
d26	2.44082321	-12.380931
D0	0.15544867	-11.880635
D1	1.07983125	-11.259776
D2	1.00312095	-11.39308
D3	0.3864102	-11.293586
D4	1.50285825	-12.11771
D5	0.93914177	-12.771049
D6	1.76652798	-12.988379
D7	0.80038338	-14.278965
D8	2.01216384	-14.06645
D9	1.96391678	-12.745121

AMH	std-dev	mean
d24	0.75887526	-10.5825
d25	0.28437065	-8.87
d26	0.3967787	-8.7333333
D0	1.87510889	-9.8166667
D1	1.47499153	-9.32
D2	0.35874782	-9.455
D3	1.7262773	-8.685
D4	1.02940274	-9.208
>D8	1.89032625	-10.866667

SOX9	std-dev	mean
d24	0.58488603	-3.1075
d25	0.90915895	-2.188
d26	0.27897132	-2.4125
D0	0.44761873	-1.58
D1	1.05136581	-2.0133333
D2	0.2753052	-1.1225
D3	0.27389195	-0.489
D4	0.44565542	-0.9
>D8	0.06819443	0.5925

SRY	std-dev	mean
d24	0.48030372	-2.4925
d25	0.40475301	-1.7475
d26	0.49013604	-1.5033333
D0	0.07094736	-0.765
D1	0.27176143	-0.44
D2	0.97589275	-4.235
D3	2.07646334	-3.86
D4	1.59695961	-4.336
>D8	1.36324368	-4.3333333

Female

FGF9	Std-Dev	mean
d26	0.59666373	-13.22248
D0	0.97999287	-11.955115
D1	1.26021659	-10.885263
D2	1.59237551	-12.26105
D3	1.39441178	-10.723046
D4	1.43975127	-13.547857
D5	1.18566393	-13.013561
D6	1.70042236	-12.69392
D7	1.44342134	-12.362655
D8	0.95537655	-12.446764
D9	1.28953986	-12.467403

FOXL2	Std-Dev	mean
d26	1.5769777	-14.772777
D0	2.56723975	-12.559378
D1	1.26719381	-10.235241
D2	2.6416806	-10.733566
D3	2.9434488	-9.9623942
D4	0.79076801	-14.424968
D5	4.42618165	-13.610474
D6	2.00363036	-12.51978
D7	0.9790437	-10.195214
D8	0.84828495	-10.24848
D9	0.97633926	-13.591147

FST	Std-Dev	mean
d26	1.04756099	-11.091936
D0	2.31735511	-10.348897
D1	1.44449287	-9.3115303
D2	2.59861574	-10.422056
D3	1.92078144	-9.6914133
D4	0.76252361	-9.3632953
D5	0.55454756	-9.8996138
D6	0.54066236	-9.5828089
D7	1.88066221	-8.5147234
D8	0.82900225	-8.1462226
D9	0.21049989	-8.9703985

RSPO1	Std-Dev	mean
d26	0.78502266	-10.615683
D0	1.48167523	-9.6727582
D1	2.62907354	-10.119049
D2	1.70292165	-8.8100917
D3	1.89872093	-9.6830324
D4	0.72470704	-14.158132
D5	2.3475652	-11.416548
D6	1.40382641	-12.407859
D7	1.82224091	-10.402456
D8	1.4449994	-10.098752
D9	0.35616634	-13.638462

WNT4	Std-Dev	mean
d26	0.66732075	-10.965275
D0	1.62307914	-9.6157953
D1	1.99537666	-9.2095446
D2	3.04693513	-10.817928
D3	1.86727987	-11.205534
D4	0.85629128	-12.051951
D5	1.50442851	-11.192795
D6	0.89343682	-11.395745
D7	1.86855863	-8.9160963
D8	1.26285402	-9.0565456
D9	0.4588566	-11.956444

AMH	Std-Dev	mean
d24	1.72109461	-11.385
d25	2.37577356	-11.855
d26	1.248706	-15.71
D0	3.14652189	-13.84
D1	2.41324643	-15.8975
D2	0.37476659	-15.875
D3	1.25299641	-14.8
D4	2.23034377	-17.576667
>D8	2.94448637	-16.9

AMH	Std-Dev	mean
d24	0.70760158	-6.41
d25	0.95661905	-4.498
d26	0.83660026	-6.385
D0	0.63042049	-6.744
D1	1.92142086	-8.36
D2	0.94752309	-5.2
D3	0.15502688	-5.0366667
D4	1.50094415	-4.885
>D8	1.02339566	-3.106
